# Assessment of Whole Grain Ancient Wheat Sourdough in Lyophilised and Native Forms for Cookie Formulation

**DOI:** 10.3390/foods13213363

**Published:** 2024-10-23

**Authors:** Nikola Maravić, Biljana Pajin, Miroslav Hadnađev, Tamara Dapčević-Hadnađev, Mladenka Pestorić, Dubravka Škrobot, Jelena Tomić

**Affiliations:** 1 Faculty of Technology, University of Novi Sad, Bul. Cara Lazara 1, 21000 Novi Sad, Serbia; pajinb@tf.uns.ac.rs; 2 Institute of Food Technology, University of Novi Sad, Bul. Cara Lazara 1, 21000 Novi Sad, Serbia; miroslav.hadnadjev@fins.uns.ac.rs (M.H.); tamara.dapcevic@fins.uns.ac.rs (T.D.-H.); mladenka.pestoric@fins.uns.ac.rs (M.P.); dubravka.skrobot@fins.uns.ac.rs (D.Š.); jelena.tomic@fins.uns.ac.rs (J.T.)

**Keywords:** ancient wheat, sourdough, cookies, rheology, sensory analysis

## Abstract

This study explored the potential of two forms of sourdough—native and lyophilised—obtained through the spontaneous fermentation of whole grain flours from ancient wheat varieties, for cookie production. The research involved evaluated the dough’s rheological properties through creep and recovery measurements and Mixolab analysis, assessing proximate composition, physical attributes, texture, colour, and sensory characteristics using the Rate-all-that-apply (RATA) method. The rheological analysis revealed that native sourdough significantly impacted dough behaviour, making it more challenging to process. Although differences were observed in the proximate composition, colour, and texture, these factors did not influence the samples as much as the rheological parameters. Sensory evaluation identified Khorasan lyophilised sourdough, along with its control sample, as the most promising, while modern wheat, spelt, and emmer exhibited potentially undesirable attributes. Based on these findings, it was concluded that lyophilised Khorasan sourdough was very favourable for cookie production and should be considered for further in-depth research and development. This suggests that the lyophilised forms of ancient wheats could offer valuable alternatives for cookie formulation, with implications for both the processing and sensory attributes of the final product.

## 1. Introduction

In the landscape of food consumption, a notable shift towards healthier dietary choices has reshaped consumer preferences. This trend has driven the food industry to innovate, addressing the growing demand for high-quality products that not only meets consumers demands, but also contributes to overall well-being. Among these commodities, bakery and fine bakery products hold a significant share of the market, offering a wide assortment of products. Therefore, these types of products provide the opportunity to create new foods with added nutritional value and to meet the diverse needs of consumers. To achieve this, a significant part of this endeavour is the exploration of alternative ingredients and innovative production methods that redefine the boundaries of traditional food manufacturing [1,2].

Within the realm of fine bakery products, the improvement of nutritional and functional properties is one of the main focuses. This involves the formulation of products enriched with proteins, fibres, and other functionally valuable components. A pivotal strategy in this pursuit is the substitution of conventional wheat flour with alternative, nutritionally rich ingredients or the use of various technological processes (fermentation, extrusion, malting, etc.) with the potential to modify the nutritional quality of the raw materials. While these approaches have potential, they can also potentially cause some complications and challenges, particularly in terms of dough processability and the preservation or change in the sensory and nutritional quality of the final product [3,4,5,6,7,8].

Among the countless technological innovations, one ancient biotechnological process—sourdough fermentation has regained its popularity, both in the scientific community, as well as in the field of the food industry, as a promising technology for the development of new food products. Basically, sourdough fermentation involves the symbiotic interaction of various microorganisms comprising lactic acid bacteria and yeasts [9]. Although sourdough fermentation is mainly associated with bread-making, currently there is a trend in its application in numerous other products as well. In recent years, sourdough fermentation has been mostly relegated to the domain of artisanal bakeries. This marginalisation resulted from its perceived inefficiencies, such as extended production times and difficulties in ensuring consistent product quality [10]. However, an exploration into the complexities of sourdough fermentation has unveiled numerous benefits, highlighting its potential for the advancement of food, such as improving the technological properties of dough, significantly enhancing product quality, and extending shelf life [11].

During the fermentation process, a series of biochemical transformations occur, fundamentally altering the composition of the dough. Lactic acid bacteria and yeasts collaboratively contribute to the development of unique properties in the final product [12]. This symbiotic interaction enhances the dough’s flavour and texture profiles, resulting in a product that provides a complex sensory experience [13]. Additionally, sourdough fermentation holds immense promise as a catalyst for nutritional enhancement. By breaking down and modifying antinutrients, such as phytic acid, sourdough fermentation unlocks the latent nutritional potential of grains, thereby enhancing the bioavailability of essential minerals [14]. Moreover, the synthesis of bioactive compounds and antioxidants during fermentation further enhances the nutritional profile of bakery products, aligning with the evolving demands of health-conscious consumers [15,16].

Considering all the aforementioned advancements, it can be concluded that sourdough fermentation offers a wide range of innovations in the food industry and possesses exceptional potential as a means for incorporating unconventional flours and underexploited cultures into bakery and fine bakery products [12]. In this regard, sourdough fermentation is recognised as an adequate technological process for the production of bakery products based on ancient wheat grains. Compared to “modern” wheat varieties, although inferior in terms of technological quality, these grains have a better nutritional profile characterised by higher protein content, soluble dietary fibres, lipids, minerals, vitamins, and bioactive compounds [17,18,19]. Ancient wheat flours are predominantly used as whole grain, whereby the higher fibre content negatively affects the rheological behaviour of the dough and, consequently, the texture and sensory properties of the products, which can be overcome by using sourdough fermentation [20]. Furthermore, ancient wheat varieties are gaining attention from the scientific community for biodiversity preservation, as they are highly adaptable to climate and require low agricultural inputs, making them extremely suitable for organic production [21]. Our literature review revealed studies investigating the effects of sourdough fermentation on the nutritional and techno-functional properties of ancient wheat flours, while our research focused on exploring the potential for sourdough fermentation from flours derived from these varieties and found that its use for creating fine bakery products is almost negligible.

The aim of this study was to explore the potential of two forms of sourdough (native and lyophilised), obtained through the process of spontaneous fermentation of whole grain flours from modern and ancient wheats (emmer—*Triticum turgidum* L. subsp. *dicoccum*, spelt—*Triticum aestivum* L. subsp. *spelta* and Khorasan—*Triticum turgidum* L. subsp. *turanicum*), in the production of cookies. This research involved a comprehensive analysis, starting with creep and recovery and a Mixolab rheological analysis of the dough. Additionally, the study evaluated the nutritional content, technological, and sensory characteristics of the final cookie products. Therefore, the influence of different forms of sourdough on the quality and acceptability of the cookies was determined. Furthermore, the insights gained from this research will provide a basis for the sample selection of further in-depth studies, as well as further directions for the development of functional and health-promoting baked goods.

## 2. Materials and Methods

Emmer, spelt, and Khorasan grains were obtained directly from a local producer, Poljoprivredno gazdinstvo Spelta Jevtić (Bačko Gradište, Republic of Serbia), while a modern wheat variety, harvested in the same year, was provided by the local milling company, Danubius d.o.o. (Novi Sad, Republic of Serbia). Prior to processing, the harvested crops underwent a six-month storage period, ensuring optimal conditions for subsequent milling. Subsequently, the hulled grains were dehulled using Heger’s large-scale friction de-huller (Herrenberg, Germany). The milling process was performed using a large-scale stone mill from Osttiroler Getreidemühlen (Dölsach, Austria). In order to obtain whole wheat flour, which served as the main ingredient in the breadmaking process, grains of the modern wheat variety underwent the milling process using a Bühler laboratory mill (Uzwil, Switzerland). Other ingredients used in the formulation of the cookies, such as vegetable fat, sugar sodium bicarbonate, ammonium bicarbonate, and sugar were purchased from local suppliers.

### 2.1. Sourdough Production

Spontaneously fermented emmer, spelt, Khorasan, and modern wheat sourdough were prepared through a backslopping procedure (every 24 h, 5 days) in a laboratory incubator (Friocell 111, MMM Medcenter Einrichtungen GmbH, München, Germany). The spontaneously fermented sourdough was prepared as is described in our previous paper published by Tomić et al. [22]. Briefly, whole wheat flour and demineralized water were mixed in a 1:1 (*w*/*w*) ratio with a resulting dough yield [(dough mass/flour mass) × 100] of 200 and incubated at 25 °C for 24 h. After the first fermentation, four additional backslopping steps were carried out by mixing a portion of the fermented dough with flour and water (fermented dough:flour:water = 1:2:2 (*w*/*w*)) at 24 h intervals. After this procedure, the sourdough reached a state known as mature sourdough, characterised by stable microflora and consistent properties. Afterwards, the refreshment procedure was repeated weekly, following the protocol of combining the mature dough with fresh flour and demineralized water in a 1:2:2 ratio (starter:flour:water). The mixture was fermented at 25 °C for 6 h, after which it was stored in a refrigerator at 4 °C until the next refreshment or product preparation. The 6 h fermentation period was determined based on findings from Tomić et al. [22], which identified this duration as optimal for achieving the desired pH level and the appropriate stage of sourdough maturation. One portion of the mature sourdough was frozen and lyophilised, while the rest of sourdough was kept refreshed and used in a fresh state in cookie formulations. The lyophilisation process was performed using a freeze dryer (Alpha 2–4 LSC plus, Christ, Osterode, Germany) on the sample that went through refreshment 6h fermentation. The samples were immediately frozen at −80 °C for 24 h to ensure complete solidification. The drying phase was conducted under a vacuum of 0.128 mbar at a shelf temperature of 20 °C for 36 h, during which sublimation of the frozen water occurred. After this process, the samples were stored in sealed bags at room temperature until further analysis.

### 2.2. Cookie Formulations

For each wheat variety (modern wheat, spelt, Khorasan, emmer), formulations of cookies were produced under the same processing conditions by substituting whole wheat flour with a certain amount of sourdough obtained from the same wheat variety in native and lyophilised forms. In the case of the native form, the sourdough was activated through a standard refreshment process before the dough was prepared. This process involved mixing mature dough with fresh flour and demineralized water in a 1:2:2 ratio (starter:flour:water) and allowing the mixture to ferment at 25 °C for 6 h. After this fermentation period, the native sourdough was utilised for cookie production.

To ensure a final dough moisture content of 24% for all the cookies, the dry matter of all the ingredients was considered in the theoretical calculations. These calculations followed a theoretical approach to determine the moisture level of the cookie dough [23]. In order to achieve an appropriate flour substitution level, special attention was given to maintain an equal proportion of dry matter from the fermented dough (approximately 25%) derived from both of the types of sourdough samples (native and lyophilised). More precisely, the lyophilised sourdough, which had approximately 1% moisture, resulted in a 25% substitution of whole wheat flour across all the samples. In contrast, the native sourdough, with moisture content ranging from 54.83% to 56.53%, achieved varying substitution levels: 57% for modern wheat and spelt sourdoughs, 55% for Khorasan sourdough, and 56% for emmer sourdough. To fulfil the conditions of the final dough’s moisture, the amount of water added varied among the samples, based on calculations. The other ingredients were used in the same proportions (sugar 15%, fat 20%, NaCl 0.5%, NaHCO_3_ 0.3%, NH_4_HCO_3_).

The dough was prepared using the following procedure: vegetable fat was mixed with sugar for 2 min, and then water was added and thoroughly blended to obtain a homogenous mixture. Finally, all the other ingredients were added together and mixed for an additional 3 min. The obtained dough was sheeted after preparation on a pilot scale dough sheeter (Macpan, Thiene, Italy) to the desired thickness (3.0 mm). The cookie samples were shaped using a circular cutter (d = 45 mm) and baked at 190 °C for 11 min in a laboratory oven (MIWE gusto^®^ CS, Arnstein, Germany). The cookies were produced in four batches where each batch yielded 20 cookies. The obtained cookies were left to cool down at room temperature for 1 h and then they were sealed in polyethylene bags and stored for further analysis.

### 2.3. Cookie Dough Analysis

#### 2.3.1. Mixolab Cookie Dough Analysis

The mixing behaviours of flour and sourdough mixes were analyzed using Mixolab (Chopin Technologies, Villeneuve-la-Garenne, France), following the standard “Chopin+” protocol [24]. The ratio of flour–lyophilizate and flour–fresh sourdough was kept as is noted in the formulation section. The protocol involved an initial mixing at 30 °C for 8 min, followed by heating to 90 °C over 15 min (at a rate of 4 °C/min), holding at 90 °C for 7 min, cooling to 50 °C over 10 min (at a rate of 4 °C/min), and then holding at 50 °C for 5 min. Throughout the process, the mixing speed was maintained at a constant 80 rpm. The Mixolab curve indicators included water absorption (WA%), which represents the percentage of water needed for the dough to achieve a torque of 1.1 Nm, and dough development time (DDT), the time required to reach the maximum torque at 30 °C. Stability refers to the amount of time for which the Mixolab curve remains within a maximum consistency of 11%, as observed while mixing. The initial maximum consistency (C1) is used to determine the maximum torque during mixing, while the minimum consistency (C2) indicates the lowest torque value during mechanical and thermal stress. The peak torque (C3) represents the maximum torque during the heating phase, and the minimum torque (C4) is the lowest torque observed during cooling to 50 °C. The breakdown torque is calculated as the difference between C3 and C4. The final torque (C5) refers to the torque after cooling to 50 °C, and the setback torque is the difference between C5 and C4.

#### 2.3.2. Creep and Recovery Analysis of Cookie Dough

A rheological analysis was conducted on freshly prepared cookie dough samples according to their provided formulations. Measurements were taken in triplicate at a temperature of 25 ± 0.1 °C using a Haake MARS rheometer (Thermo Scientific, Karlsruhe, Germany). To prevent sample slippage, serrated parallel-plate geometry with a 35 mm diameter was used, maintaining a constant gap of 1 mm for all the tests. Once a sample was loaded, the excess dough was trimmed, covered with paraffin oil to prevent drying, and allowed to rest for 300 s to release any residual stress from loading. Creep measurements were taken at a shear stress of 10 Pa for 180 s, followed by a recovery phase at 0 Pa for 420 s. The primary parameter monitored was the maximum creep compliance (J_max_).

### 2.4. Cookie Analysis

#### 2.4.1. Chemical Composition of Cookies

The proximate composition of the raw materials and cookies, including protein (AOAC method 920.87), fat (AOAC method 922.06), ash (AOAC method No. 923.03) and moisture mass fractions (AOAC method No. 925.09), was determined by AOAC standard methods of analysis [25]. The total dietary fibre content of the obtained cookies was determined using the Megazyme Total Dietary Fiber Assay Kit (Neogen, Lansing, MI, USA) using a method adopted from AOAC method 985.29 and AACC method 32-07. The available carbohydrate content was obtained by subtracting the sum of mass of water, protein, fat, ash and dietary fibre in g per 100 g of sample. The energy value was determined according to the European Regulation No. 1169/2011 [26]. 

#### 2.4.2. Physical Properties and Colour of the Cookies

Cookies taken randomly from the batch were used for the measurements of physical parameters, including the mass (g), the diameters of the baked crackers (d_1_ and d_2_, perpendicular to each other), and the thicknesses of the cookies (h), by a Vernier calliper. Eccentricity was calculated as the ratio between the diameters (Equation (1)). Spread factor was calculated as the ratio between the average diameters and the average thickness (Equation (2)).

(1) $$Eccentricity=\frac{d_{1}}{d_{2}}$$

(2) $$Spread\;factor=\frac{d_{average}}{h}$$

where *d_average_* was calculated as the average value of cookie diameters d_1_ and d_2_.

The colour of the cookies’ top surface was measured 24 h after baking using a Chroma Meter Minolta CR-400 (Konica Minolta Co., Osaka, Japan). Colour measurements were made on five randomly selected cookies per sample batch and the results were expressed as L* (lightness/darkness), a* (redness/greenness), and b* (yellowness/blueness).

#### 2.4.3. Textural Properties of Cookies

The cookies’ hardness and fracturability were measured using a TA-XT2 Texture Analyser (Stable Micro Systems, Godalming, UK) equipped with a 30 kg load cell and three-point bending rig (HDP/3PB). The two adjustable supports of the rig base plate were placed 30 mm apart so as to support the sample. The upper blade was positioned to be equi-distant from the two lower supports. While measuring, the upper blade descended at a speed of 1 mm/s until a contact force of 50 g was detected, and then travelled a distance of 5 mm through the cookies at a speed of 3.0 mm/s. Measurements were performed 24 h after baking in six replicates per batch at ambient temperature ((25 ± 1) °C).

#### 2.4.4. Sensory Evaluation

A sensory evaluation of the cookies was performed by FINS in Novi Sad with a panel of 15 trained assessors experienced in general and cookie-related sensory attributes. Prior to testing, sensory experts from FINS developed a lexicon of sensory attributes related to cookies’ products by reviewing the literature, adding new terms, and ensuring that the descriptors were non-hedonic and non-redundant. The sensory attributes were selected using the Rate-All-That-Apply (RATA) method, which was applied to the cookie samples. The assessors were instructed to select all the relevant attributes/descriptors from a list and rate their intensity on a scale from 0 to 5 (with 0 indicating that the attribute is not present; 1—very weakly noticeable attribute to 5—extremely noticeable attribute). A list of 38 sensory attributes was compiled for characterising the individual cookie samples, categorised into 9 visual, 5 olfactory, 13 texture, 3 taste, and 8 flavour descriptors (Table 1). Each evaluation session lasted 90 min, divided into two 45 min sub-sessions, conducted in two replicates, with the order of the samples randomised and the samples identified by three-digit codes. The panellists rinsed their mouths with mineral water between samples.

This study was approved by the Ethics Committee of the Institute of Food Technology in Novi Sad, University of Novi Sad, Serbia (Ref. No. 24-52-3).

### 2.5. Statistical Analysis

All the measurements were performed at least in triplicates if not stated differently. The mean values of the replicates for the analysed parameters were statistically processed using the software package XLSTAT version 2023.3.1 (Addinsoft, New York, NY, USA). An analysis of variance (ANOVA) and a Tuckey’s honest significant difference test (*p* < 0.05) were used to determine the significance of the differences between the sample mean values.

## 3. Results and Discussion

### 3.1. Cookie Dough Evaluation

#### 3.1.1. Dough Rheological Behaviour

**Figure 1:**
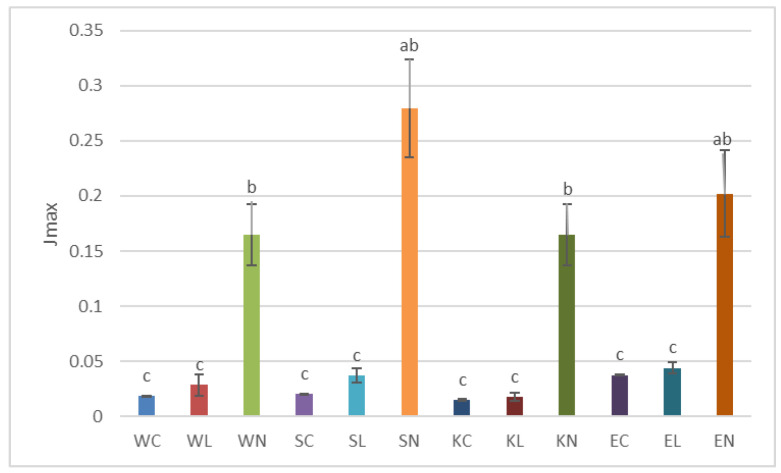
Rheological properties of cookie dough (maximum creep compliance, J_max_); W—modern wheat; S—spelt; K—Khorasan; E—emmer; C—control; L—lyophilised sourdough addition; N—fresh sourdough addition. Mean values in histogram with the same lower case letter are significantly different according to the ANOVA followed by the Tukey’s test (*p* < 0.05).

The creep test was used to determine the effect of sourdough type substitution on the rheological changes in the cookie dough. The maximum creep compliance (J_max_) value, obtained from the creep-recovery curve at the conclusion of the creep phase under constant shear stress, was used to quantify the dough softness, where higher J_max_ values indicated high dough extensibility [27]. The results of the test are presented in Figure 1.

Substituting the flour with lyophilised sourdough did not influence the dough rheology, showing a slight but not significant increase compared to the control sample from the same wheat variety. The highest values of the J_max_ value between the lyophilised sourdough-containing samples were identified in the emmer variety. On the other hand, in all the samples where native sourdough was added, a significant increase in J_max_ was observed, indicating that the dough became significantly softer. Among native sourdough-containing samples, SN exhibited the highest J_max_ values, followed by EN, KN, and WN, respectively.

The explanation for these observations can be related to the fact that fermentation influences the breakdown of large protein aggregates responsible for dough’s structural integrity into smaller aggregates, contributing to system softening and a decrease in elasticity [28]. Protein structure degradation can be attributed to proteolytic activity and a reduction in disulphide bonds. Additionally, in an acidic environment, proteins carry a positive net charge, leading to increased intramolecular electrostatic repulsion forces and the consequent unfolding of gluten proteins, which increases their solubility and weakens their structure [29,30]. In our previous study by Tomić et al. [22], we observed that the pH of the tested sourdough samples ranged from 4.2 to 4.4, indicating that its addition resulted in a more acidic environment. Furthermore, the metabolic activity of the microflora in mature sourdough, stored between refreshments at 4 °C, still persists, although the process occurs very slowly. With the introduction of mature sourdough in the refreshment process, it could be assumed that the protein structure of the whole system is more deteriorated compared to the sourdough in the lyophilised form, where microbial and enzymatic activity is halted during storage. Additionally, it has to be taken into account that the lyophilisation process reduces microbial viability (Supplementary Table S1) [31,32] which can reflect on the ongoing fermentation during processing, while native sourdough, with higher microbial viability, undergoes more active fermentation, leading to greater proteolytic activity and more intensive protein breakdown. This results in softer dough, as shown by the higher J_max_ values.

Houben et al. [33] investigated the rheological behaviour of amaranth dough with the addition of different lactobacilli strains and pointed out that those strains influenced dough differently, which could also be attributed to their metabolic activity. The previously mentioned factors and results of our previous study [22], where the ratio of the yeasts to lactic acid bacteria varied among the different sourdoughs, along with the wheat variety used and the differences in the sourdough forms, can possibly explain the differences among the samples. Therefore, it can be concluded that the addition of native sourdough can result in cookie dough that is more difficult to process, indicating the possibility of modifying processing parameters or formulation.

#### 3.1.2. Mixolab Analysis

**Figure 2:**
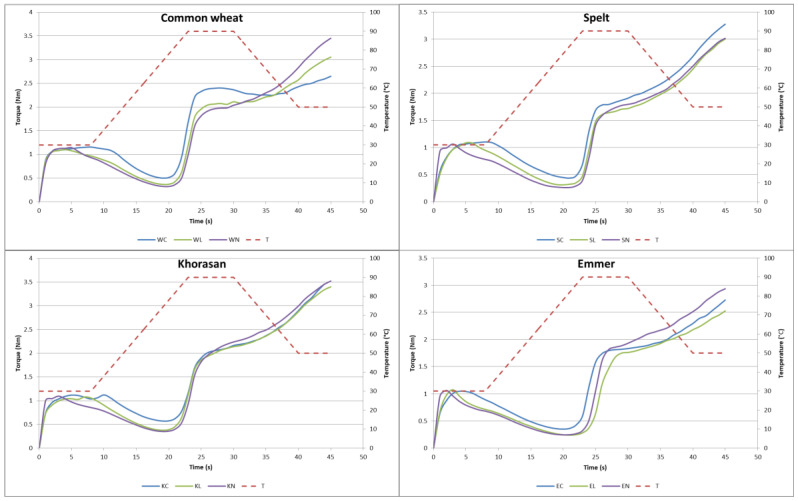
Mixolab curves of tested cookie dough samples; W—modern wheat; S—spelt; K—Khorasan; E—emmer; C—control; L—lyophilised sourdough addition; N—fresh sourdough addition.

The Mixolab curves of all the samples are presented in Figure 2. The Mixolab analysis detailed in this study offers a comprehensive evaluation of dough properties, allowing us to compare the effects of lyophilised and native sourdough addition.

According to Mixolab measurements, it can be concluded that the addition of sourdough influenced the dough’s behaviour significantly in most of the parameters (Figure 2). The water absorption varied among the tested wheat varieties, as did the type of sourdough addition, being in a range of 51.95% for control spelt flour-based samples (SCs) to 60.5% for Khorasan samples containing 25% of native sourdough (KNs). There is no consistent trend in influence via the addition of freeze dried and native sourdough. In the samples made with modern wheat and Khorasan, the addition of lyophilised sourdough led to a decrease in water absorption, whereas native sourdough had no effect on this parameter. Conversely, in samples made with spelt and emmer, the addition of native sourdough significantly increased the water absorption. The lyophilised sourdough increased the water absorption in spelt but had no effect in the case of emmer. Only for the spelt sample are the findings consistent with Codina et al. [34], who assessed the Mixolab properties of adding dry sourdough (1–5%) to modern wheat flour, while all the other cases are the opposite.

In the initial phase of Mixolab mixing (parameters’ dough development time and stability), the similar tendency of these values to decrease can be noticed with the addition of sourdough. These results are in agreement with previously mentioned research by Codina et al. [34], who suggested that this trend is probably a consequence of the intensified hydrolysis of proteins and starch, which leads to the release of water from the gluten system, which affects the dough’s consistency and stability. The addition of native sourdough results in a more pronounced decrease in these parameters, indicating even less stable and weaker dough. This can be attributed to increased proteolytic activity in native sourdough compared to lyophilised sourdough samples (Supplementary Table S1). The creep and recovery analyses presented in this paper confirmed this trend for native sourdough, but not for lyophilised dough. The differences in the results obtained by these two rheological methods could be related to the fact that, in the creep and recovery measurements of the cookie dough, all the ingredients were examined, while in the Mixolab tests, only the water, flour, and sourdough in the same ratios as they were in the formulations were tested.

In addition to these results, the data obtained from the Mixolab tests also give us valuable insights into the behaviour of proteins (C2), starch gelatinisation (C3), starch gel stability (C4), and retrogradation (C5) [35,36]. As can be seen from the obtained data, there is a trend in the decrease in the C2 value which indicates a weaker protein structure with more pronounced lower values in the case of native sourdough addition in all the wheat variety cases. This can be attributed to the higher enzymatic activity that occurs with sourdough addition, which is especially pronounced with native sourdough. Again, a difference between the native and freeze-dried forms is noted and can be most likely attributed to the freeze-drying process, which may reduce enzymatic activity, leading to lower overall activity in the lyophilised form [32,37].

During the heating phase, protein denatures and releases water, while starch granules start to absorb water and swell leading to starch gelatinisation and, consequently, an increase in viscosity. According to the obtained Mixolab curves, it can be concluded that only the wheat control samples without sourdough addition had pronounced peak torque (C3 value), followed by a slight decrease in their C4 value. However, in the rest of the tested samples, the values of the parameter C3 were not pronounced, meaning that there was a constant increase in the measured torque, so the values of the C4 parameter were higher compared to the C3 values. This could be related to the combined effect of starch gelatinisation, protein denaturation, and enzymatic activity. Namely, during the further heating, the proteins were denaturing and forming a network contributing to the structural integrity and increased resistance of the dough. High protein content along with a strong gluten network can therefore significantly influence resistance during heating. Moreover, this phenomenon could also be ascribed to delayed or extended starch gelatinisation, meaning that starch gelatinises over a broader temperature range, resulting in a more gradual increase in the torque values rather than a sharp peak at the C3 point. Delayed gelatinisation could be a consequence of higher soluble dietary fibre (SDF) content [38]. Since the cookie samples with the addition of sourdough were characterised by higher content of SDF, the presence of SDF could also be one of explanations for this phenomenon. Finally, if the enzymatic activity is low, starch gel is more stable for heating and mixing and the protein network can have more impact on increased torque values.

### 3.2. Cookie Evaluation

#### 3.2.1. Proximate Composition of Cookies

The proximate composition analysis of the cookies, as detailed in Table 2, reveals significant nutritional differences attributable to the wheat varieties and the type of sourdough addition.

The results for the protein content varied across the tested samples, with the emmer group registering the highest protein levels, followed by spelt. This highlights the higher protein content of these two varieties of wheat, which can significantly enhance the nutritional value of bakery products. This has been confirmed by our previous work where flours of these wheat varieties were analysed and showed the same trend [39]. However, there is other research that shows that Khorasan and spelt are richer in proteins [40]; therefore, this could be attributed to the specific varieties and the various conditions of cultivation. Additionally, the ash content was particularly elevated in the spelt and emmer group, suggesting that these ancient grains contribute to increased mineral density in baked goods. The cookie samples differed substantially in TDF, from 7.12% to 9.26%, where the cookies produced from modern wheat and Khorasan in general had higher TDF contents. Since all the cookie samples contained more than 6 g of fibre per 100 g, they can also be labelled “high in fibre” according to Regulation (EC) No 1924/2006 [41]. Regarding the influence of sourdough addition on the nutritional composition of the final products, it can be observed that native sourdough samples demonstrate nutritional profiles that closely align with those observed in the samples with lyophilizate. The most prominent changes were obtained in the moisture and soluble fibre content.

Notably, the moisture content in the samples with native sourdough was generally lower compared to their controls, while the samples with lyophilizates were more similar. Regarding the soluble fibre content, the cookies with sourdough in both forms in the case of wheat and spelt had significantly higher content of soluble dietary fibres. Recent research shows that sourdough fermentation significantly impacts the chemical and physical properties of fibre. In regard to the higher acidity in sourdough, the higher content of SDF could be the result of a higher hydrolytic solubilisation effect in certain endogenous enzymes. Namely, Subaşi and Ercan [42], who investigated the influence of different percentages of sourdough on the different nutritional parameters of whole wheat bread, reported an increase in SDF content as a consequence of sourdough fermentation. An increase in fibre solubilisation was also shown by Păucean et al. [43] in the breads made of the ancient wheat (einkorn, spelt, emmer) flour type with the addition of sourdough produced by fermentation with the *Lactiplantibacillus plantarum* ATCC 8014 strain.

Taking into account the higher soluble fibre content in the above-mentioned samples with sourdough, it can be assumed that higher moisture loss during baking could be a consequence of the changes in the structures of the proteins, starches, and fibres. In addition, the surface area of the cookies is larger relative to their volume, which increases the potential for moisture loss during baking. The higher soluble fibre content in sourdough contributes to the initial higher water retention in the dough, but during baking, the presence of sourdough could exacerbate the moisture loss as a result of the complex interactions between the dough’s altered structures. The disparity in moisture loss across the cookie samples made from various flour varieties can be attributed to the distinct structural components of each flour, as well as the extent to which these components are altered during fermentation.

#### 3.2.2. Physical, Colour, and Textural Properties of Cookies

**Figure 3:**
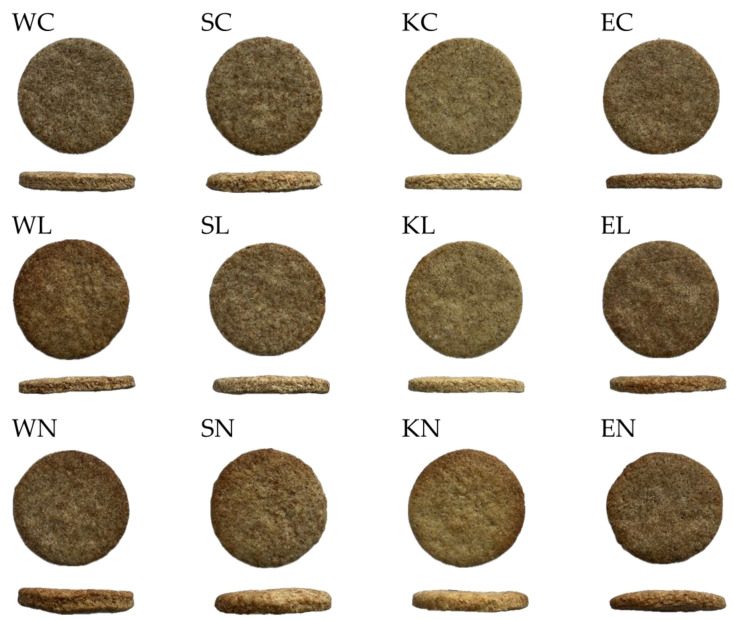
Images of produced cookies; W—modern wheat; S—spelt; K—Khorasan; E—emmer; C—control; L—lyophilised sourdough addition; N—fresh sourdough addition.

The assessment of the colour, physical, and textural properties of the cookies presented in Figure 3 made from various samples, as detailed in Table 3, provides an in-depth understanding of how different types of sourdough addition influence the final product’s quality. The colour of the cookies showed significant variations among the samples. Specifically, the lightness (L*) values were the highest in all the Khorasan samples and the lowest in all the emmer samples, with the WN sample exhibiting similar lightness to the emmer samples. Within each wheat variety group, there were no significant differences in lightness, indicating that lightness is primarily a characteristic of the wheat variety itself, with the addition of sourdough having no impact. Regarding the values a* and b*, all the samples containing native sourdough, except for spelt and emmer in the b* parameter, showed significantly higher values. Since higher a* and b* values indicate stronger red and yellow colour components, this increase can be attributed to intensified Maillard reactions and caramelisation during baking, which are likely due to the higher presence of reaction precursors resulting from sourdough fermentation [44,45].

Along with the colour, other physical attributes, such as texture and shape, are vital in determining the overall quality of the cookies. The eccentricity values, consistently close to 1 across all the samples, indicate a uniform, circular shape. However, the height and spread factor reveal that the addition of native sourdough significantly reduces spreading during baking, resulting in thicker cookies. These findings align with the study by Alioğlu and Özülkü [46], which also observed a decrease in the spread factor of biscuits when sourdough was included in the formulation. Additionally, the incorporation of native sourdough significantly reduced the weight of the cookies compared to the control samples. The physical properties of the cookies are influenced by various factors, including protein, lipid, and fibre content, water-binding capacity, and the processes occurring during baking [47]. Considering that sourdough fermentation leads to changes in protein and starch [48], these differences can be attributed to the fermentation process. These changes also impact the textural properties of the cookies.

Specifically, the hardness of all the cookies with native sourdough addition showed a significant decrease, while lyophilised sourdough addition had no significant impact, except in the case of spelt, where lower hardness values were also noted. In addition to the changes in the cookies’ constituents that occurred during fermentation, gas was produced either through fermentation or via the chemical reaction between the leavening agents like sodium bicarbonate and lactic acid, which caused the expansion of the initially incorporated air bubbles during mixing. Consequently, the size and distribution of these bubbles indirectly affected their characteristics such as hardness [49]. Furthermore, the addition of native sourdough significantly impacted the fracturability of the cookies, resulting in higher values. Similar to this study, Sahin et al. [50] demonstrated that cookies with sourdough exhibited significantly lower firmness. This finding was also confirmed by Alioğlu and Özülkü [46], although their results were closer to the control, and the difference was less pronounced.

#### 3.2.3. Sensory Properties of Cookies

The data obtained using the RATA method were processed through various approaches and presented in different ways, complementing each other to provide a clearer picture of the influence of the two forms of sourdough (native and lyophilised) on the sensory profile of the cookies.

**Figure 4:**
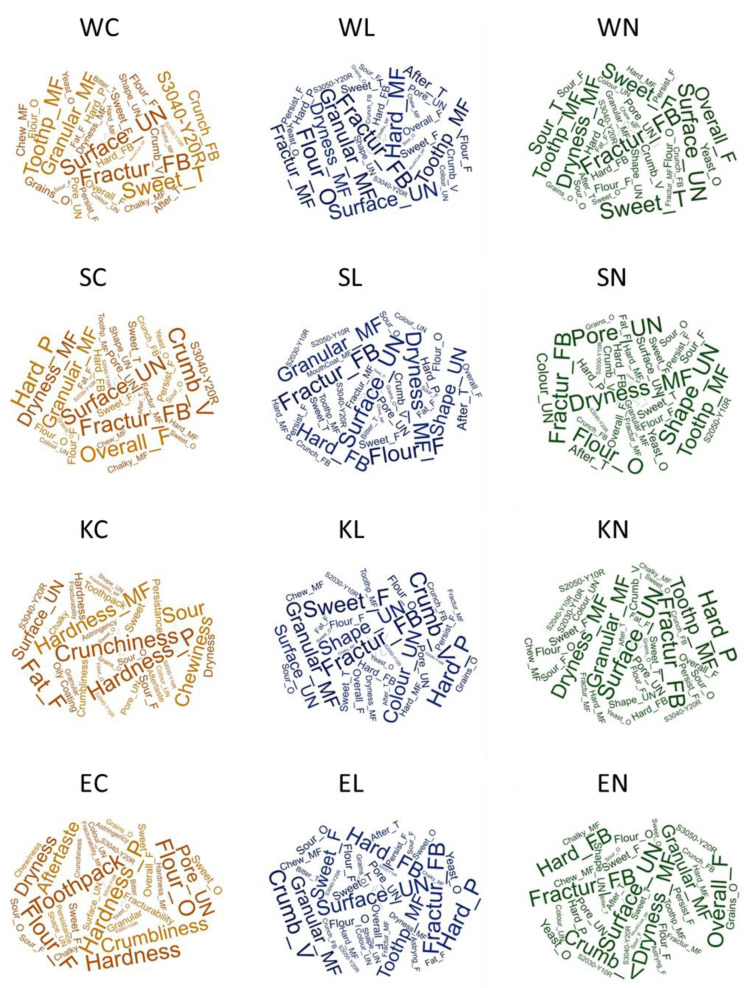
Word clouds showing the profile of the samples within each wheat variety group based on attribute frequency. W—modern wheat; S—spelt; K—Khorasan; E—emmer; C—control; L—lyophilised sourdough addition; N—fresh sourdough addition.

For presenting sensory characteristics, a “word cloud” was used as a visual representation, where the size of each word reflected its frequency or importance (Figure 4). It highlighted key terms and provided an intuitive overview of the main themes or attributes, making it easier to grasp the overall sensory profile at a glance.

Venn diagrams were used to visually represent the unique and shared sensory attributes among the cookie samples. Each circle represented a group of samples with common characteristics, while overlaps showed shared attributes and non-overlapping areas revealed unique traits. This method helped clarify how the cookie samples differed from and resembled each other.

Additionally, spider graphs were utilised to depict the attributes’ intensities and frequencies. The sum of the intensity values was multiplied by their corresponding frequencies [51], yielding a weighted measure that reflected both the frequency of perception and the perceived strength of each attribute. This provided a nuanced interpretation of the sensory profiles associated with each sample.

Another important result of the RATA method was the graphical representation of products and attributes. This visualisation clearly showed how different sensory characteristics related and contrasted, aiding in the interpretation of complex sensory data. The purpose of this was to capture and compare the sensory profiles effectively, making it easier to identify key patterns and distinctions between the products.

The most commonly mentioned attributes across all the samples pertained to textural attributes, such as hardness, fracturability, crumbliness, and mouthfeel attributes. Attributes related to the visual aspects of the cookies, such as shape unevenness, surface unevenness and pore unevenness, were frequently mentioned across all the sample groups, indicating the significant impact of flour treatment on the visual appeal of cookies. For instance, the samples SN and DN exhibited higher frequencies of mentions for these attributes, suggesting notable visual distinctiveness that could impact consumer appeal.

Colour attributes, represented by colour codes, are not so visible in the Figure since they are distributed among samples. These attributes are more clearly illustrated in the Venn diagram, where differences and similarities are shown. This may be attributed to the specific flour types or processing methods that affect Maillard reactions and caramelisation during baking, thereby impacting the colour profile of the cookies.

Other attributes such as yeast and grain odour, or sweet and sour taste showed variability among the samples. Notably, a yeasty odour distinguished the samples WN and KN due to their native sourdough content. While attributes like sweet odour and bitter taste were less frequently mentioned, indicating a lesser impact on the overall sensory experience, their presence highlights the diversity in flavour profiles across the cookie types. This variation highlights the complex interactions between ingredient composition and sensory perception, which could be highly important from the perspective of consumer preference.

**Figure 5:**
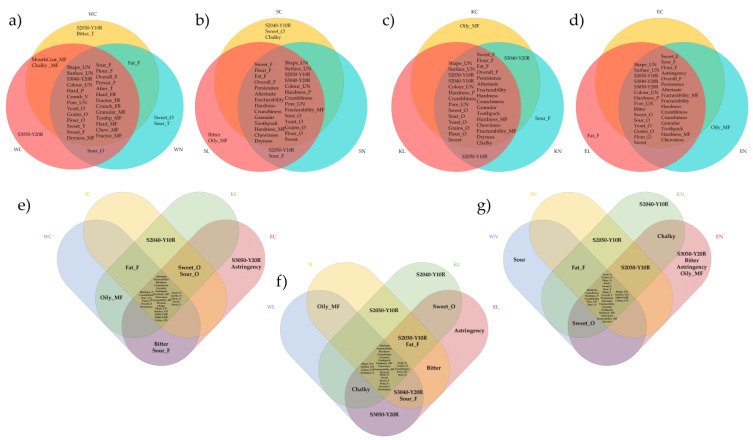
Venn diagram related to the attributes selected by 30% of the panel to describe the cookies: (**a**) wheat-based; (**b**) spelt-based; (**c**) Khorasan-based; (**d**) emmer-based; (**e**) all control samples; (**f**) samples with lyophilised sourdough; (**g**) samples with native sourdough; W—modern wheat; S—spelt, K—Khorasan; E—emmer; C—control; L—lyophilised sourdough addition; N—fresh sourdough addition.

Analysing RATA data with Venn diagrams (Figure 5) showed that several high- and medium-frequency attributes were common to the cookie samples, while certain unique traits distinguished them. This analysis, combined with insights from the word clouds, provided valuable information about the unique and overlapping sensory characteristics. At first glance of the Figure, it can be seen that most of the attributes were common to all the samples. What differentiated them were colour nuance and some specific flavour, odour, or taste attributes. For some samples, there was nothing specific to the sample; their attributes were already found in other samples.

Besides attributes that could ‘positively’ contribute to samples, there were also some other parameters that were undesirable in the product. For instance, a bitter taste and astringency were attributes that are not commonly associated with this type of product and that were likely characterised negatively. A bitter taste was noted by panellists in all the emmer samples, as well as in the modern wheat control and in the sample with lyophilised spelt sourdough. Additionally, astringency was observed in all the emmer samples.

**Figure 6:**
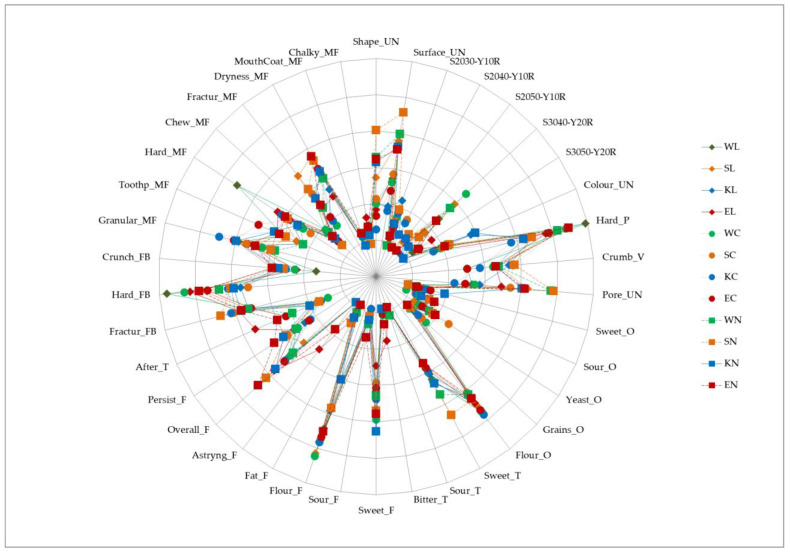
Spider plot with intensity and frequencies; W—modern wheat; S—spelt; K—Khorasan; E—emmer; C—control; L—lyophilized sourdough addition; N—fresh sourdough addition.

After analysing qualitative data presented via word clouds and Venn diagrams, spider graphs were created and are shown in Figure 6 to fully capture the data. These graphs effectively illustrate the complex interplay of sensory attributes across the entire range of cookie samples, highlighting the intensity of each attribute. This descriptive analysis provided a detailed understanding of the sensory attributes of each cookie variant, allowing for a nuanced comparison across the different formulations and treatments.

With this approach, the attributes and samples that stand out can be identified more precisely, which might not have been as clear in the previous section. For instance, visual attributes such as shape unevenness, surface unevenness, and pore unevenness were prominently noted among the samples, while the most pronounced were in samples where native sourdough was used. As already mentioned, colour nuances distinguished the samples. However, when intensities were taken into consideration, a complete overview of this aspect was obtained, revealing how prominently each specific nuance was presented. Textural attributes such as hardness, fracturability, crumbliness, and granular mouthfeel were consistently high across most of the samples, although their values varied among the samples.

A sweet odour was more pronounced in the emmer and Khorasan samples, while the odour of the grains was least emphasised in the emmer samples. Comparing the whole wheat flour odour in the samples, it can be concluded that the addition of native sourdough reduced this sensation. Sweetness was more pronounced when native sourdough was used with wheat, spelt, and Khorasan, whereas a bitter taste was notably higher in all the emmer samples compared to others.

**Figure 7:**
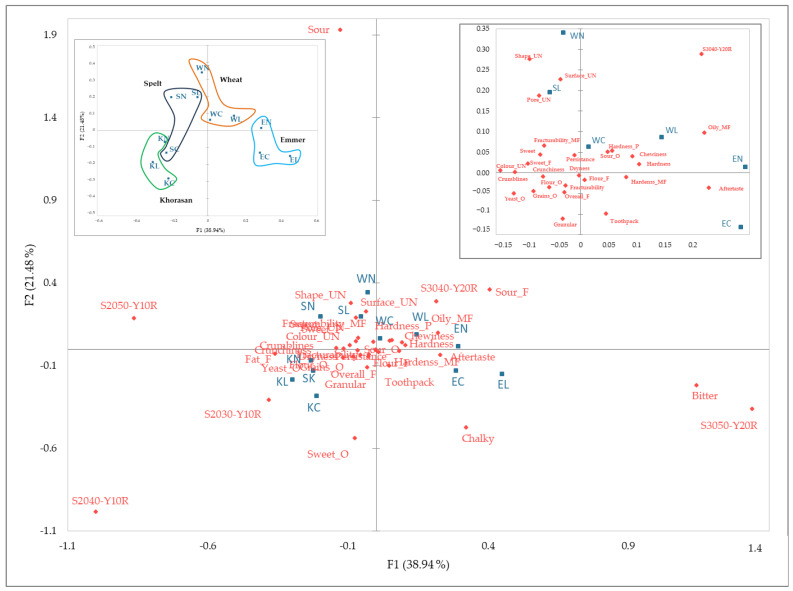
Biplot of cookie samples with attribute differentiation using RATA method; W—modern wheat; S—spelt; K—Khorasan; E—emmer; C—control; L—lyophilised sourdough addition; N—fresh sourdough addition.

Another important result of the RATA feature was the graphical representation of the products and attributes, as shown in Figure 7. In the subsequent chart, it could be observed that the first two axes accounted for 60.56% of the inertia, which might have suggested that these two axes were sufficient for the analysis. After eliminating the least mentioned 30%, 38 attributes remained, with 29 showing significant differences (*p* < 0.05) that could discriminate between the cookie samples. This method has been used by other authors in studies of different matrices, such as non-wheat flour biscuits [52], wine [53], and tea [54]. All of these studies have demonstrated that the RATA method is a suitable and reliable methodology for describing samples and provides a valuable alternative to conventional descriptive analysis for gathering information about the sensory perception of a product.

As seen in Figure 6, the samples were differentiated and grouped. The grouping by the wheat variety was more pronounced compared to the grouping by the type of sourdough substitution. Colour was the attribute that was distinguished among the samples, while emmer samples were characterised by bitter taste and astringency. The sour taste was most pronounced in the WN sample, while sourness was an attribute of the native sourdough samples that was also evidently present the in wheat and spelt samples. On the other hand, a sweet odour mostly characterised the Khorasan samples.

## 4. Conclusions

The results of this study demonstrate that lyophilised sourdough has greater potential for use in cookie production compared to its native form. Although there were notable differences in the nutritive, physical, colour, and textural properties of the cookies, the most significant variations were observed in the dough rheology tests and sensory evaluation. Specifically, rheology measurements revealed distinctly different behaviours that could have led to challenges during dough processing, suggesting the need for adjustments in formulations and process parameters. At the same time, a sensory evaluation focusing on attributes likely to negatively impact consumer perception, such as bitter taste and astringency, resulted in the exclusion of certain samples, including all emmer samples, the spelt-lyophilised sample, and the wheat control sample. In conclusion, the Khorasan cookie sample with lyophilised sourdough and its corresponding control exhibited the highest potential for further detailed evaluation, the investigation of different substitution levels, and comparison.

## Figures and Tables

**Table 1:** The list of attributes with codes used for sensory evaluation.

Attribute (Modality)	Descriptors	Abbreviation	Definition
**Appearance** (*Visual)*	Shape unevenness	Shape_UN	The amount of curve present when the cookies are placed on a flat surface and the cross-section is viewed from the side.
	Surface unevenness	Surface_UN	The degree of uniformity perceived on the surface of the cookies: the grade of the uniformity on the distribution cells.
	Colour		The sensation produced on the cookies’ colour, resulting from the stimulation of the retina by light waves in the visible region of the spectrum: the nuance of the typical cookies’ colour. These colours were visually classified according to the NCS System^®^
	S2030-Y10R	S2030-Y10R	
	S2040-Y10R	S2040-Y10R	
	S2050-Y10R	S2050-Y10R	
	S3040-Y20R	S3040-Y20R	
	S3050-Y20R	S3050-Y20R	
	Colour unevenness	Colour_UN	The uniformity of the baked colour on the whole surface of the cookies: the areas are different from the typical cookies’ colour.
	Pore unevenness	Pore_UN	The unevenness of the pores on the transverse fracture of the cookies: the grade uniformity on the distribution cell.
**Texture—Manual** *(Palpatory)*	Crumbliness	Crumb_V	Falls apart into small crumbs or powder shortly after manipulation.
	Hardness	Hard_P	Manual force is required to break or separate the sample into pieces.
**Odour** (*Olfactory*)	Sweet	Sweet_O	A sweet odour that evokes the impression of sugar or fruit.
	Sour	Sour_O	An odour characteristic of the dough having been fermented using a traditional sourdough.
	Yeast	Yeast_O	A special odour similar to that of the yeast powder or the dough fermented with yeast.
	Grains	Grains_O	The intensity of the odour is associated with wheat grains.
	Flour	Flour_O	The intensity of the odour is associated with whole wheat flour.
**Taste** (*Gustatory*)	Sweet	Sweet_T	A fundamental taste factor of which sucrose is typical.
	Sour	Sour_T	A fundamental taste associated with a citric acid solution.
	Bitter	Bitter_T	A basic taste factor of which caffeine is typical.
**Flavour** (*Olfactory, gustatory and trigeminal sensations in the mouth*)	Sweetness	Sweet_F	A sweet aroma that evokes impression of sugar or fruit.
	Sourness	Sour_F	An aroma characteristic of the dough having been fermented using a traditional sourdough.
	Whole wheat flavour	Flour_F	The intensity of the flavour is associated with whole wheat flour.
	Vegetable fat/oil	Fat_F	The intensity of the flavour is associated with vegetable fat/oil.
	Overall flavour intensity	Overall_F	An overall product rating that takes into account how well the present characteristic notes match the product, their intensity, the origin of the identifiable aroma, and the blend/balance of the aroma.
	Astringency	Astryng_F	The feeling of a puckering or a tingling sensation on the surface and/or edges of the tongue and mouth.
	Persistence	Persist_F	The duration of the olfato-gustatory sensation that is perceived after the bolus leaves the mouth.
	Aftertaste	After_T	An olfactory and/or gustatory sensation that occurs after removing the product and that differs from the sensation that occurred while the product was in the mouth.
**Texture—Initial Bite** (*Tactile*)	Hardness	Hard_FB	The cookies withstand substantial force on the initial bite.
	Fracturability	Fractur_FB	The cookies easily break apart, forming a loose piece in the mouth with the first bite.
	Crunchiness	Crunch_FB	The sound and force with which the sample breaks and cracks, with a crunchy sound being heard.
**Texture—Mouthfeel** (*Oral)*	Hardness	Hard_MF	The force/work required to compress the sample between the teeth.
	Fracturability	Fractur_MF	The degree to which the samples fracture when compressed between the molar teeth and chewed evenly, measuring how much the sample fractures.
	Chewiness	Chew_MF	The mechanical textural property relating to the number of chews necessary to bring the product to the state necessary for swallowing.
	Toothpack	Toothp_MF	The degree to which the sample sticks to the surface of the teeth.
	Dryness/Moisture absorbency (Dryness)	Dryness_MF	This term describes how well a material stays dry or how well it absorbs moisture: the absorption of saliva and moisture in the mouth while chewing.
	Oily Mouth Coating	MouthCoat_MF	A smooth, grease-like coating perceived on the roof of the mouth after swallowing.
	Granular	Granular_MF	A sensation during mastication caused by the presence of product granules.
	Chalky	Chalky_MF	The measure of a dry, powdery sensation in the mouth after swallowing.

**Table 2:** Proximate composition of cookies.

Sample	Moisture	Ash	Protein	Fat	Insoluble Fiber	Soluble Fiber	Carbohydrates	Energy (kJ/kcal)
WC	4.59 ± 0.03 ^b^	1.45 ± 0.01 ^c^	8.98 ± 0.06 ^de^	15.57 ± 0.03 ^c^	7.19 ± 0.36 ^abc^	1.07 ± 0.01 ^f^	62.23 ± 0.26 ^a^	1853/441
WL	4.47 ± 0.06 ^b^	1.44 ± 0.11 ^c^	9.43 ± 0.18 ^d^	16.46 ± 0.11 ^c^	7.63 ± 0.16 ^ab^	1.32 ± 0.02 ^cde^	60.58 ± 0.24 ^bc^	1870/446
WN	3.93 ± 0.11 ^c^	1.50 ± 0.03 ^bc^	9.47 ± 0.30 ^d^	16.75 ± 0.28 ^b^	7.92 ± 0.26 ^a^	1.34 ± 0.01 ^bcde^	60.43 ± 0.35 ^d^	1882/449
SC	5.09 ± 0.01 ^a^	1.75 ± 0.01 ^a^	11.4 ± 0.23 ^bc^	15.75 ± 0.04 ^c^	6.03 ± 0.23 ^d^	1.09 ± 0.05 ^f^	60.00 ± 0.50 ^c^	1854/442
SL	5.03 ± 0.11 ^a^	1.73 ± 0.08 ^a^	10.99 ± 0.13 ^c^	15.86 ± 0.19 ^c^	5.96 ± 0.28 ^d^	1.38 ± 0.07 ^bcd^	60.44 ± 0.41 ^c^	1856/443
SN	4.49 ± 0.06 ^b^	1.74 ± 0.06 ^a^	10.81 ± 0.20 ^c^	16.02 ± 0.24 ^ab^	5.90 ± 0.27 ^d^	1.51 ± 0.06 ^ab^	61.04 ± 0.05 ^d^	1873/446
KC	5.10 ± 0.06 ^a^	1.44 ± 0.04 ^c^	9.57 ± 0.45 ^d^	16.17 ± 0.21 ^c^	6.47 ± 0.06 ^cd^	1.17 ± 0.05 ^ef^	61.26 ± 0.08 ^abc^	1864/444
KL	4.96 ± 0.10 ^a^	1.47 ± 0.02 ^c^	8.95 ± 0.20 ^de^	16.02 ± 0.11 ^c^	6.79 ± 0.23 ^bcd^	1.33 ± 0.02 ^cde^	61.81 ± 0.04 ^ab^	1857/443
KN	4.48 ± 0.03 ^b^	1.50 ± 0.03 ^bc^	8.29 ± 0.23 ^e^	16.14 ± 0.45 ^a^	6.79 ± 0.11 ^bcd^	1.24 ± 0.02 ^def^	62.80 ± 0.52 ^d^	1870/446
EC	5.21 ± 0.09 ^a^	1.80 ± 0.01 ^a^	12.62 ± 0.16 ^a^	16.17 ± 0.11 ^c^	6.00 ± 0.09 ^d^	1.44 ± 0.07 ^abc^	58.21 ± 0.14 ^d^	1862/444
EL	5.05 ± 0.04 ^a^	1.69 ± 0.00 ^ab^	12.04 ± 0.26 ^ab^	16.37 ± 0.35 ^c^	6.31 ± 0.34 ^cd^	1.35 ± 0.01 ^bcd^	58.55 ± 0.47 ^d^	1867/445
EN	4.56 ± 0.03 ^b^	1.75 ± 0.05 ^a^	11.98 ± 0.2 ^ab^	16.28 ± 0.46 ^ab^	6.01 ± 0.27 ^d^	1.60 ± 0.05 ^a^	59.43 ± 0.46 ^e^	1877/447

W—modern wheat; S—spelt; K—Khorasan; E—emmer; C—control; L—lyophilised sourdough addition; N—fresh sourdough addition. The mean values ± standard deviation in the same column are significantly different (*p* < 0.05) if they are followed by the same letters in the superscript.

**Table 3:** Colour, physical, and textural properties of cookies.

Sample	Colour Properties			Physical Properties				Textural Properties	
	L* (D65)	a* (D65)	b* (D65)	Weight (g)	Height (mm)	Eccentricity	Spread Factor	Hardness (g)	Fracturability (mm)
WC	60.3 ± 1.6 ^bc^	8.5 ± 0.9 ^bcd^	24.7 ± 2 ^de^	5.75 ± 0.10 ^a^	5.53 ± 0.26 ^cde^	0.99 ± 0.02 ^a^	7.62 ± 0.38 ^c^	2983.87 ± 162.63 ^ab^	36.46 ± 0.40 ^cdef^
WL	59.2 ± 1.1 ^c^	9.1 ± 0.4 ^b^	25.8 ± 0.7 ^cd^	5.29 ± 0.12 ^bc^	5.38 ± 0.14 ^def^	1.01 ± 0.03 ^a^	7.74 ± 0.24 ^bc^	2672.36 ± 157.45 ^abc^	36.51 ± 0.20 ^cdef^
WN	57.1 ± 1.1 ^de^	10.6 ± 0.8 ^a^	27.7 ± 1.1 ^b^	5.22 ± 0.28 ^c^	6.88 ± 0.56 ^a^	1.02 ± 0.04 ^a^	6.16 ± 0.60 ^e^	2444.25 ± 289.79 ^cd^	38.16 ± 0.44 ^ab^
SC	60.7 ± 1.2 ^bc^	7.8 ± 0.6 ^de^	23.7 ± 0.6 ^ef^	5.75 ± 0.15 ^a^	5.96 ± 0.25 ^bc^	0.99 ± 0.03 ^a^	7.30 ± 0.26 ^c^	3125.77 ± 187.23 ^a^	37.11 ± 0.20 ^cd^
SL	61.0 ± 1.6 ^b^	8.0 ± 0.4 ^cde^	23.6 ± 0.7 ^ef^	5.56 ± 0.05 ^abc^	5.67 ± 0.12 ^bcd^	1.00 ± 0.03 ^a^	7.59 ± 0.14 ^c^	2094.78 ± 117.38 ^d^	36.72 ± 0.50 ^cdef^
SN	60.7 ± 1.0 ^bc^	8.6 ± 0.6 ^bcd^	27 ± 1.5 ^bc^	5.12 ± 0.18 ^c^	6.85 ± 0.16 ^a^	1.01 ± 0.03 ^a^	6.48 ± 0.23 ^de^	2142.17 ± 140.33 ^d^	38.35 ± 0.28 ^a^
KC	66.4 ± 0.9 ^a^	5.8 ± 0.3 ^f^	27 ± 0.7 ^bc^	5.73 ± 0.51 ^ab^	4.85 ± 0.29 ^f^	1.00 ± 0.01 ^a^	9.00 ± 0.54 ^a^	2301.42 ± 112.42 ^cd^	36.1 ± 0.43 ^ef^
KL	67.2 ± 1.0 ^a^	6.2 ± 0.5 ^f^	28.1 ± 0.7 ^b^	5.33 ± 0.16 ^abc^	4.88 ± 0.12 ^f^	1.00 ± 0.00 ^a^	8.88 ± 0.18 ^a^	2116.47 ± 28.56 ^d^	36.17 ± 0.33 ^def^
KN	65.7 ± 2.0 ^a^	7.2 ± 1.6 ^e^	30.7 ± 1.4 ^a^	5.17 ± 0.17 ^c^	6.07 ± 0.21 ^bc^	1.00 ± 0.02 ^a^	7.22 ± 0.28 ^cd^	1268.25 ± 238.00 ^e^	37.28 ± 0.35 ^bc^
EC	56.3 ± 1.4 ^de^	8.7 ± 0.5 ^bc^	23.2 ± 0.9 ^f^	5.49 ± 0.16 ^abc^	5.13 ± 0.25 ^def^	1.01 ± 0.01 ^a^	8.49 ± 0.45 ^ab^	2672.05 ± 92.88 ^abc^	36.27 ± 0.25 ^def^
EL	55.5 ± 1.1 ^e^	9.1 ± 0.4 ^b^	24.1 ± 0.5 ^ef^	5.44 ± 0.10 ^abc^	5.07 ± 0.16 ^ef^	1.00 ± 0.03 ^a^	8.57 ± 0.25 ^a^	2541.13 ± 236.97 ^bcd^	36.01 ± 0.07 ^f^
EN	57.3 ± 1.1 ^d^	8.9 ± 0.5 ^b^	25.8 ± 1.2 ^cd^	5.10 ± 0.13 ^c^	6.15 ± 0.28 ^b^	0.99 ± 0.02 ^a^	7.25 ± 0.32 ^c^	2147.79 ± 175.87 ^d^	37.01 ± 0.28 ^cde^

W—modern wheat; S—spelt; K—Khorasan; E—emmer; C—control; L—lyophilised sourdough addition; N—fresh sourdough addition. The mean values ± standard deviation in the same column are significantly different (*p* < 0.05) if they are followed by the same letters in the superscript.

## Data Availability

The original contributions presented in this study are included in the article/Supplementary Materials; further inquiries can be directed to the corresponding author.

## Supplementary Materials

The following supporting information can be downloaded at: https://www.mdpi.com/article/10.3390/foods13213363/s1, Supplementary Table S1. Microbiological screening of sourdough in native form and after freeze-drying, expressed as ratio of Native/Freeze Dried (%).
