# Supplementary material for: Assessment of Whole Grain Ancient Wheat Sourdough in Lyophilised and Native Forms for Cookie Formulation
Source: Foods. 2024 Oct 23;13(21):3363. doi: 10.3390/foods13213363 (PMC11545800; doi:10.3390/foods13213363)
Supplement: Supplementary file 1 [file foods-13-03363-s001.zip › foods-3225582-supplementary.pdf]

Supplementary Table S1. Microbiological screening of sourdough in native form and after freeze-drying, expressed as ratio of Native/Freeze Dried (%)

	Sample	Modern wheat	Spelt	Emmer	Khorasan
Yeast	Native	$1.4*10^7 \pm 6*10^6$	$6.33*10^7 \pm 9.45*10^7$	$4.32*10^7 \pm 5.8*10^6$	$5.17*10^7 \pm 1.23*10^7$
	Freeze dried	$2.58*10^6 \pm 5.3*10^5$	$7.25*10^6 \pm 8.32*10^5$	$7.54*10^6 \pm 3.4*10^5$	$7.42*10^6 \pm 2.69*10^6$
	Survival rate %	18.43	11.45	22.24	14.35
Lactobacilli	Native	$2.45*10^9 \pm 2.93*10^8$	$3.39*10^9 \pm 1.19*10^8$	$3.39*10^9 \pm 1.94*10^8$	$2.66*10^9 \pm 2.31*10^7$
	Freeze dried	$6.32*10^8 \pm 3.75*10^7$	$8.12*10^8 \pm 7.23*10^7$	$7.15*10^8 \pm 4.4*10^7$	$5.12*10^8 \pm 1.25*10^7$
	Survival rate %	25.80	23.95	21.09	19.25
